# Direct‐to‐consumer genetic testing for factor V Leiden and prothrombin 20210G>A: the consumer experience

**DOI:** 10.1002/mgg3.1468

**Published:** 2020-09-16

**Authors:** Sarah L. Elson, Nicholas A. Furlotte, Bethann S. Hromatka, Catherine H. Wilson, Joanna L. Mountain, Helen M. Rowbotham, Elizabeth A. Varga, Uta Francke

**Affiliations:** ^1^ 23andMe, Inc Sunnyvale CA USA; ^2^ Nationwide Children's Hospital Columbus OH USA; ^3^ Department of Genetics Stanford University Stanford CA USA; ^4^ Present address: Puma Biotechnology South San Francisco CA USA

## Abstract

**Background:**

Clinical genetic testing for inherited predisposition to venous thromboembolism (VTE) is common among patients and their families. However, there is incomplete consensus about which individuals should receive testing, and the relative risks and benefits.

**Methods:**

We assessed outcomes of receiving direct‐to‐consumer (DTC) results for the two most common genetic risk factors for VTE, factor V Leiden in the *F5* gene (FVL) and prothrombin 20210G>A in the *F2* gene (PT). Two thousand three hundred fifty‐four customers (1244 variant‐positive and 1110 variant‐negative individuals) of the personal genetics company 23andMe, Inc., who had received results online for *F5* and *F2* variants, participated in an online survey‐based study. Participants responded to questions about perception of VTE risk, discussion of results with healthcare providers (HCPs) and recommendations received, actions taken to control risk, emotional responses to receiving risk results, and perceived value of the information.

**Results:**

Most participants (90% of variant‐positive individuals, 99% of variant‐negative individuals) had not previously been tested for *F5* and/or *F2* variants. The majority of variant‐positive individuals correctly perceived that they were at higher than average risk for developing VTE. These individuals reported moderate rates of discussing results with HCPs (41%); receiving prevention advice from HCPs (31%), and making behavioral changes to control risk (e.g., exercising more, 30%). A minority (36%) of variant‐positive individuals worried more after receiving VTE results. Nevertheless, most participants reported that knowing their risk had been an advantage (78% variant‐positive and 58% variant‐negative) and were satisfied knowing their genetic probability for VTE (81% variant‐positive and 67% variant‐negative).

**Conclusion:**

Consumers reported moderate rates of behavioral change and perceived personal benefit from receiving DTC genetic results for VTE risk.

## Abstract 2

We assessed the consumer experience receiving direct‐to‐consumer (DTC) genetic risk for venous thromboembolism (VTE). 2354 customers (1244 variant‐positive and 1110 variant‐negative individuals) of the personal genetics company 23andMe, Inc., who had received results online for risk variants in clotting factor genes F2 and F5, participated in an online survey‐based study. Participants responded to questions about perception of VTE risk, discussion of results with healthcare providers and recommendations received, actions taken to control risk, emotional responses to receiving risk results, and perceived value of the information.

## 1 INTRODUCTION

Venous thromboembolism (VTE) is characterized by deep vein thrombosis (DVT), pulmonary embolism (PE), or both. VTE affects 300,000–600,000 individuals in the United States each year, can cause long‐term complications and is associated with high morbidity and mortality (Beckman, Hooper, Critchley, & Ortel, 2010; Behravesh et al., 2017). Genetic, acquired, and situational factors influence risk for VTE.

The two most common genetic risk factors for inherited predisposition (thrombophilia) are factor V Leiden (c.1691G>A, p.Arg506Gln) in the *F5* gene (FVL), found in up to 25% of people with VTE (Kujovich, 1993a, 2011), and prothrombin 20210G>A (c.*97G>A) in the *F2* gene (PT) (Kujovich, 1993c). Both heterozygotes and homozygotes are at elevated risk for VTE. Odds ratios for first time thrombosis for FVL heterozygotes and homozygotes are approximately 5 and 11, respectively; odds ratios for PT heterozygotes and homozygotes are approximately 3 and 11 (Gohil, Peck, & Sharma, 2009; Simone et al., 2013; Zhang et al., 2018).

Risk is compounded by numerous acquired and situational factors, including oral contraceptive use, hormone replacement therapy (HRT), pregnancy, and prolonged periods of inactivity, among others (Kujovich, 1993b, 1993c; Varga & Kujovich, 2012). Management of risk in asymptomatic individuals may include avoiding compounding risk factors and/or temporary prophylaxis. For instance, known thrombophilia risk may influence decisions about contraception and HRT and may prompt increased vigilance and/or prophylaxis during pregnancy and postpartum (American College of Obstetricians & Gynecologists Women's Health Care Physicians, 2013; Bates et al., 2012; Kujovich, 1993c; Trenor et al., 2011).

There is incomplete agreement about which individuals should be tested for these two variants to inform treatment and prevention decisions (De Stefano & Rossi, 2013; Zhang et al., 2018). Clinical guidelines such as those of the Evaluation of Genomic Applications in Practice and Prevention Working Group recommend testing in circumstances where test results are likely to influence clinical management and discourage routine testing for patients with VTE and their family members (Evaluation of Genomic Applications in Practice and Prevention (EGAPP) Working Group, 2011; Grody, Griffin, Taylor, Korf, & Heit, 2001; Kujovich, 1993c; Stevens et al., 2016). Other guidelines diverge, and in practice, clinical genetic testing in these patients and their families is widespread (Stevens et al., 2016; Varga & Kujovich, 2012). There have been public health efforts to raise awareness of VTE, including, in 2008, the U.S. Surgeon General's recommendation to raise consumer awareness about inherited thrombophilia (Beckman et al., 2010; Office of the Surgeon General (US) & National Heart, Lung, and Blood Institute (US), 2008), and there are indications of consumer interest in understanding inherited risk (Hellmann, Leslie, & Moll, 2003).

Given the apparent gap between clinical guidelines and consumer and public health organizations’ interest in greater awareness, it is useful to assess the impact of direct‐to‐consumer (DTC) testing for thrombophilia. For over 10 years 23andMe, Inc., a personal genetics company, has offered DTC results for numerous genetic risk factors, including select variants associated with thrombophilia (Carere et al., 2014; Francke et al., 2013).

Here, we report data from a nine‐month study on consumer response to DTC genetic testing for FVL and PT. We discuss recall of test results, sharing of test results with health care providers (HCPs) and family members, HCP recommendations, behavioral changes based on test results, and emotional responses to testing.

## 2 METHODS

### 2.1 Ethical compliance

All participants in the study provided consent and answered surveys online according to 23andMe's research protocol, which was approved by Ethical & Independent Review Services, an AAHRPP‐accredited institutional review board. Participants whose free‐text responses are quoted gave 23andMe explicit permission to do so. The study conforms to U.S. Federal Policy for the Protection of Human Subjects.

### 2.2 Recruitment

Study participants were customers of 23andMe who obtained 23andMe's personal genomic services, including a VTE genetic risk report, between November 2007 and November 2013 (Carere et al., 2014). An example of the VTE report offered during this time is in Figure S1.

23andMe research participants who were over the age of 30, had logged into their 23andMe.com account within the two‐year period prior to November 2013, were not part of any other 23andMe disease research study, and had opted to receive health results were invited to participate. Eight thousand five hundred thirty‐six variant‐positive individuals with one or more FVL or PT variant and 11,353 variant‐negative individuals were invited to participate.

### 2.3 Data collection

Study participation consisted of responding to at least one of two online surveys. Survey 1 was offered to participants from March to April 2015 and included up to 102 questions, depending on survey branching logic. Survey 1 addressed participants’ recall of the genetic test result and subsequent perception of VTE risk; personal and family history of VTE, including prior testing for FVL and PT; discussing results with HCPs and family; behavioral changes in response to test results; emotional responses to testing; and demographics. Survey 2 was offered nine months later, from December 2015 to January 2016, to participants who had taken Survey 1 and consisted of up to 51 questions. Survey 2 further interrogated participants’ health outcomes, attitudes, and understanding of the causes and consequences of VTE. Both surveys asked whether participants had viewed their 23andMe VTE report—Survey 1, if they had ever viewed the report; Survey 2, if they had viewed it within the previous nine months. Survey questions are provided in Supplemental Material.

One thousand two hundred forty‐four variant‐positive and 1110 variant‐negative individuals consented and responded to Survey 1. Seven hundred fifty‐one cases and 574 controls took both surveys (Table 1). Survey response rates, stratified by sex and variant status, are provided in Table 1 and Table S1. Rates of response were roughly consistent with previously reported rates (Tung et al., 2011).

**TABLE 1:** Characteristics of the study population

	Cases			Controls		
	Female	Male	Total	Female	Male	Total
Survey 1 contacted (N)	3961	4575	8536	5273	6080	11,353
Survey 1 responded (N)	680	564	1244	581	529	1110
Survey 1 response rate (%)	17.2%	12.3%	14.6%	11.0%	8.7%	9.8%
Took survey 1 and reported prior history of VTE (%)	8.3%	8.8%	8.5%	2.9%	2.7%	2.8%
Average age of participants who took survey 1 (years)	57.4	56.5	57.0	58.2	55.9	57.1
Survey 2 contacted (N)	617	470	1087	495	434	929
Survey 2 responded (N)	404	347	751	316	258	574
Survey 2 response rate (%)	65.5%	73.8%	69.1%	63.8%	59.4%	61.8%
Took survey 2 and reported prior history of VTE (%)	10.6%	8.1%	9.5%	1.9%	3.5%	2.6%
Average age of participants who completed survey 2 (years)	56.8	57.1	57.0	59.4	57.7	58.6
Reported having viewed 23andMe VTE report before taking Survey 1 (%)	79.8%			45.8%		
Reported having viewed 23andMe VTE report in 9 months prior to taking Survey 2 (%)	42.6%			20.4%		
Reported having prior knowledge of genetic risk for VTE (%)	9.8%			1.0%		

Highest level of education completed				
	Cases (*n* = 1,112)		Controls (*n* = 943)	
High school diploma or equivalent, or less education.	25	2.2%	26	2.8%
Some college but no degree	126	11.3%	118	12.5%
Associate college degree	68	6.1%	42	4.4%
Bachelor's college degree (for example: BA, AB, BS)	311	28.0%	284	30.1%
Master's degree (for example: MA, MS, MEng, Med, MSW, MBA)	306	27.5%	262	27.8%
Professional school degree (for example: MD, DDS, DVM, LLB, JD)	96	8.6%	75	7.9%
Doctorate degree (for example: PhD, EdD)	155	13.9%	117	12.4%
Other	25	2.2%	19	2.0%

Household annual income				
	Cases (*n* = 1,110)		Controls (*n* = 944)	
$14,999 or less	10	0.9%	11	1.2%
$15,000 to $24,999	17	1.5%	22	2.3%
$25,000 to $39,999	41	3.7%	46	4.9%
$40,000 to $59,999	88	7.9%	84	8.9%
$60,000 to $89,999	156	14.1%	136	14.4%
$90,000 to $124,999	169	15.2%	142	15.0%
$125,000 or more	449	40.5%	341	36.1%
I'd rather not say	180	16.2%	162	17.1%

Self‐reported ancestry/ethnicity				
	Cases (*n* = 1207)		Controls (*n* = 1068)	
European or White	1156	95.8%	1002	93.8%
Native American or Alaska Native	49	4.1%	57	5.3%
Latino or Hispanic	40	3.3%	39	3.7%
Middle Eastern or North African	33	2.7%	22	2.1%
Asian	25	2.1%	28	2.6%
African American or Black	13	1.1%	17	1.6%
Sub‐Saharan African	13	1.1%	13	1.2%
Pacific Islander or Oceanian	2	0.2%	2	0.2%
Other	12	1.0%	11	1.0%
I'm not sure	5	0.4%	8	0.7%

### 2.4 Data analysis

The data were analyzed by variant‐positive versus variant‐negative status and sometimes stratified by genotype and sex. Table 1, Tables S1–S3 include data from all participants who provided the relevant responses. Tables 2, 3, 4, 5, 6, 7, 8 and Table S3 include data only from participants who reported having viewed their 23andMe VTE report prior to study participation; these participants were asked to not refer to their 23andMe reports when responding. Tables 1, 3, 4, 5, 7A, 8, and Tables S1–S3 include data from Survey 1; Tables 1, 2, 6, 7B, and Table S1 include data from Survey 2. Free text responses were read and coded by two authors (SLE and HMR). Recurring themes identified in at least five responses were reported; other themes were added to the “Other” category (Table 8).

**TABLE 2:** Participant perceptions of personal VTE risk

Compared to others of the same sex and ethnicity, what is your chance of developing VTE during your lifetime?						
	1 mutation (*n* = 593)		2 mutations (*n* = 29)		Controls (*n* = 273)	
Lower	16	2.7%	2	6.9%	53	19.4%
Average	36	6.1%	0	0.0%	75	27.5%
Higher	490	82.6%	26	89.7%	51	18.7%
I'm not sure	51	8.6%	1	3.4%	94	34.4%

What are the results of your genetic testing for genes associated with VTE?						
	1 mutation (*n* = 593)		2 mutations (*n* = 29)		Controls (*n* = 272)	
I have one or more mutations that increase the risk of VTE	440	74.2%	25	86.2%	42	15.4%
I do not have any mutations that increase the risk of VTE	17	2.9%	1	3.4%	70	25.7%
I'm not sure	136	22.9%	3	10.3%	160	58.8%

**TABLE 3:** Discussion of results with others

Since you received your 23andMe results, with whom have you discussed your genetic risk for VTE? Please check all that apply.				
	Cases (*n* = 910)		Controls (*n* = 456)	
Spouse or significant other	548	60.2%	100	21.9%
Mother	304	33.4%	26	5.7%
Sister(s)	285	31.3%	26	5.7%
Child(ren)	229	25.2%	27	5.9%
Brother(s)	222	24.4%	16	3.5%
Father	181	19.9%	15	3.3%
Cousin(s)	79	8.7%	5	1.1%
Aunt(s)	51	5.6%	1	0.2%
Uncle(s)	33	3.6%	0	0.0%
Grandparent(s)	14	1.5%	0	0.0%
Friend(s)	306	33.6%	32	7.0%
Health care provider(s)	377	41.4%	32	7.0%
Online forum (23andMe or other)	39	4.3%	3	0.7%
None of the above	141	15.5%	306	67.1%

With which health care providers have you discussed your genetic risk for VTE? Please check all that apply.				
	Cases (*n* = 376)		Controls (*n* = 31)	
Primary Care Physician	333	88.1%	24	66.7%
Obstetrician/gynecologist	88	34.4% a	7	33.3%^a^
Hematologist	68	18.0%	0	0.0%
Surgeon	67	17.7%	3	8.3%
Nurse	41	10.8%	0	0.0%
Nurse Practitioner	39	10.3%	0	0.0%
Physician Assistant	34	9.0%	2	5.6%
Pharmacist	21	5.6%	0	0.0%
Genetic Counselor	15	4.0%	1	2.8%
Medical Geneticist	5	1.3%	0	0.0%
Other	53	14.0%	2	5.6%
None of the above	4	1.1%	2	5.6%

a Calculated for female respondents only.

**TABLE 4:** HCP recommendations

When you shared your 23andMe results with your healthcare provider(s), did he/she recommend any changes to your lifestyle, any changes to your medications, or additional testing?				
	Cases (*n* = 375)		Controls (*n* = 35)	
Yes	117	31.2%	5	14.3%
No	258	68.8%	30	85.7%

What did your health care provider recommend? Please check all that apply		
	Cases (*n* = 117)	
Wear compression socks/stockings	42	35.9%
Exercise more	40	34.2%
Lose weight	33	28.2%
Repeat *F5* and *F2* testing in a clinical laboratory	25	21.4%
Discontinue or change estrogen‐containing contraceptive	17	18.7%^a^
Take medication to prevent blood clots	21	17.9%
Use a blood thinner (anticoagulant) such as heparin or warfarin after surgery	20	17.1%
Suggest relatives have testing for clotting disorders	17	14.5%
Discontinue or change hormone replacement therapy	12	13.2%^a^
Have testing for other clotting disorders	15	12.8%
Use a blood thinner (anticoagulant) such as heparin or warfarin for a longer duration	13	11.1%
Use heparin during pregnancy	6	6.6%^a^
Stop smoking	6	5.1%
Other or None of the above	36	30.8%

a Calculated for female respondents only.

**TABLE 5:** Behavioral and medication changes

Have you made any of the following changes as a result of your 23andMe report on genetic risk for VTE? Please check all that apply.				
	Cases (*n* = 911)		Controls (*n* = 457)	
Exercise more	270	29.6%	56	12.3%
Took steps to lose weight	227	24.9%	48	10.5%
Started wearing compression socks/stockings	110	12.1%	7	1.5%
Discontinued or changed estrogen‐containing contraceptive	42	4.6%	1	0.2%
Took a blood thinner (anticoagulant) such as heparin or warfarin for a longer duration	35	3.8%	1	0.2%
Discontinued or changed hormone replacement therapy	34	3.7%	2	0.4%
Took a blood thinner (anticoagulant) such as heparin or warfarin after surgery	28	3.1%	1	0.2%
Stopped smoking	19	2.1%	3	0.7%
Started taking medication to prevent blood clots	18	2.0%	1	0.2%
Took a blood thinner (anticoagulant) such as heparin during pregnancy	7	0.8%	0	0.0%
Other	156	17.1%	28	6.1%
Made no changes	388	42.6%	360	78.8%

**TABLE 6:** Family testing for thrombophilia

Which of your family members has had testing for genetic mutations associated with VTE? Please check all that apply.				
	Cases (*n* = 737)		Controls (*n* = 545)	
Child(ren)	114	15.5%	34	6.2%
Sister(s)	85	11.5%	17	3.1%
Mother	76	10.3%	25	4.6%
Father	63	8.5%	26	4.8%
Brother(s)	56	7.6%	16	2.9%
Cousin(s)	26	3.5%	12	2.2%
Aunt(s)	13	1.8%	2	0.4%
Uncle(s)	8	1.1%	3	0.6%
Grandparent(s)	5	0.7%	4	0.7%
Spouse or significant other	92	12.5%	47	8.6%
Other	27	3.7%	10	1.8%
None of the above	444	60.2%	429	78.7%

Did any of your relatives get genetic testing because of your results?				
	Cases (*n* = 294)		Controls (*n* = 116)	
Yes	82	27.9%	3	2.6%
No	189	64.3%	109	94.0%
I'm not sure	23	7.8%	4	3.4%

Where were your relatives genetically tested?		
	Cases (*n* = 82)	
23andMe	39	47.6%
Through a health care provider	28	34.1%
A combination of 23andMe and health care providers	14	17.1%
I'm not sure	1	1.2%

**TABLE 7:** Perceptions of value and emotional responses to receiving VTE test results

A				
Are you satisfied or unsatisfied that you know your genetic probability for VTE?				
	Cases (*n* = 906)		Controls (*n* = 447)	
Satisfied	735	81.1%	300	67.1%
Unsatisfied	11	1.2%	6	1.3%
Neither satisfied nor unsatisfied	159	17.5%	141	31.5%

Has knowing your genetic probability for VTE been an advantage or disadvantage for you?				
	Cases (*n* = 903)		Controls (*n* = 444)	
Advantage	703	77.9%	259	58.3%
Disadvantage	1	0.1%	0	0.0%
Neither an advantage nor disadvantage	199	22.0%	185	41.7%

Do you think that you can take steps to reduce the probability of VTE?				
	Cases (*n* = 904)		Controls (*n* = 445)	
No	20	2.2%	21	4.7%
I'm not sure	102	11.3%	47	10.6%
Yes	782	86.5%	377	84.7%

Does knowing your genetic probability for VTE make you worry more or worry less about developing VTE?				
	Cases (*n* = 899)		Controls (*n* = 443)	
Worry less	105	11.7%	158	35.7%
Worry more	327	36.3%	37	8.4%
Worry same	467	51.9%	248	56.0%

B				
If you could do it all over again, would you choose to learn your genetic risk for VTE again?				
	Cases (*n* = 610)		Controls (*n* = 263)	
No	5	0.8%	4	1.5%
I'm not sure	15	2.5%	13	4.9%
Yes	590	96.7%	246	93.5%

**TABLE 8:** Positive and negative impacts of knowing genetic risk for VTE

	Theme	% reporting	Sample response
Cases reporting positive impact (*n* = 642)	Increased knowledge of VTE and/or personal risk	16.7%	"Awareness of the condition is always valuable."
	Ability to recognize signs/symptoms	16.5%	"I had the VTE episode without remembering my 23 and Me genetic report. After it was diagnosed, I checked the report and discovered the higher risk I was at. If I had remembered the report, I probably would have realized what my symptoms meant sooner and so would my PCP."
	Ability to take preventative measures	63.6%	"Because I am aware, I was able to avoid estrogen containing hormone replacement therapy and learned how to prevent VTE on long flights."
	Ability to inform HCP	24.9%	"I feel that being informed is a positive. When I had surgery, the entire team knew of my VTE risks and I was monitored closely for signs of a problem. They made sure I had compression stockings and the leg compressors during and after my surgery although I decided not to have any meds. I felt that I was covered with the Team knowing about my condition and taking steps to prevent any problems from occurring."
	Sharing risk information with family	9.3%	"23 & Me has undoubtedly saved my life, my children's life and my brother's life. … My daughter was on the strongest estrogen containing birth control and was smoking when we found out my results. She was tested and also has the VTE risk and immediately saw her doctor and changed birth control and stopped smoking...."
	Other	5.0%	N/A
Cases reporting negative impact (*n* = 201)	Potential for or actual increased worry	68.2%	"I worry a little more. But I think the awareness and preventive measures I practice outweigh the worry."
	Unpleasantness of knowing risk	9.5%	"Being aware that you are more prone to something bad is disappointing."
	Potential for or actual unnecessary medical care	5.0%	"Being overly sensitive to 'symptoms' that might lead to a false alarm and a needless trip to medical providers."
	Negative reaction from HCP after sharing 23andMe results	6.5%	"My healthcare provider didn't know what to make of it or how to consider your results."
	Negative impact on family, or negative emotions about family risk	7.5%	"I am sorry I passed the genetic risk down to my son who has already had a problem develop."
	Other	8.5%	N/A
Controls reporting positive impact (*n* = 253)	Increased knowledge of VTE and/or personal risk	19.4%	"It's helpful to know the contribution that genetics has to VTE."
	Ability to recognize signs/symptoms	7.5%	"I will be more informed about what causes it and also the symptoms."
	Ability to take preventative measures	35.6%	"Since my genetics predict an incidence of VTE near the average, there's not a great deal of new information available. However, being aware of the existence of VTE has caused me to consider working standing and stretching breaks into my day and when I travel."
	Ability to inform HCP	14.2%	"If I needed surgery, I would advise my physician of this test result."
	Sharing risk information with family	2.0%	"The positive impact of knowing about my risk for VTE was that my grandmother died from it, so just being able to share with my parents and sibs that the risk shows up in my own DNA let us all have a discussion about it."
	Satisfaction with knowing not at elevated risk	21.7%	"It's a comfort knowing that neither I nor my children have the (so far identified) genetic markers for VTE."
	Other	15.4%	N/A
Controls reporting negative impact (*n* = 47)	Potential for or actual increased worry	46.8%	"I worry since I've had several surgeries over the past two years that I could have developed a clot and not know it."
	Potential for false sense of security	12.8%	"Risk of reduced vigilance."
	Other	36.2%	N/A

## 3 RESULTS

### 3.1 Study population

The study included 1244 variant‐positive (cases) and 1110 variant‐negative individuals (controls). Among cases, 794 (64%) were FVL heterozygotes, 21 (2%) were FVL homozygotes, 401 (32%) were PT heterozygotes, three (0.2%) were PT homozygotes, 24 (2%) were FVL/PT double heterozygotes, and one (0.1%) was homozygous for FVL and heterozygous for PT.

Fifty‐four percent of study participants were female. Participants were primarily of European ancestry and had an average age of 57; most were at least college‐educated and were relatively high‐income earners (Table 1).

Eighty percent of cases and 46% of controls reported having viewed their 23andMe VTE report before taking Survey 1. A minority of participants (10% of cases and 1% of controls) knew their genetic risk for VTE prior to receiving their 23andMe genetic test results (Table 1). Among those who had had prior testing, it was often obtained because of a personal or family history of VTE (Table S2).

The surveys included questions about personal and family history of VTE. Approximately 9% of cases and 3% of controls who took Survey 1 reported a personal history of VTE. Twenty‐three percent of cases and 13% of controls reported having a first‐degree biological relative who had been diagnosed with VTE.

### 3.2 Test results and risk perception

Participants who had viewed their 23andMe VTE report prior to study participation responded to questions about their 23andMe results and VTE risk. Depending on genotype, 83%–90% of cases correctly reported that their genetic risk for VTE was higher than average, and 74%–86% of cases correctly responded that they had at least one risk variant (Table 2). In contrast, 47% of controls correctly responded that their risk was average or below average (34% were unsure), and 26% of controls correctly responded that they did not have a risk variant (59% were unsure). (Of note, the VTE report relevant to this study indicated slightly higher than average VTE risk for individuals with non‐O blood types. Forty‐five of the 51 controls who reported higher risk for VTE, and 36 of the 42 controls who responded that they had one or more mutations, had received a report that indicated slightly elevated risk due to non‐O blood type). Sixty‐nine percent of cases with two risk variants (FVL or PT homozygotes, FVL/PT double heterozygotes) correctly responded that their lifetime risk for VTE was greater than 40%, and 55% of cases with one risk variant correctly reported that their lifetime risk for VTE was 10%–40% (Table S3). Most cases and controls were unable to recall their specific genotypes.

### 3.3 Discussing results with family, friends, and HCPs

More cases than controls reported discussing their 23andMe results with their family, friends, and HCPs (Table 3). Sixty‐five percent of cases and 17% of controls discussed their results with a first‐degree family member. Sixty percent of cases and 22% of controls, discussed with a spouse or significant other.

Forty‐one percent of cases and 7% of controls discussed their results with a HCP. Among cases who discussed with a physician, most discussed with a primary care physician (88%), followed by other specialties. Cases discussed their results with fewer non‐physician providers than physicians (Table 3).

### 3.4 HCP recommendations

Thirty‐one percent of cases and 14% of controls reported that HCPs recommended changes in lifestyle or medications, or additional testing (Table 4). For cases, the most common recommendations were to wear compression socks (36%), exercise more (34%), and lose weight (28%). Smoking cessation was recommended to 5% of cases and none of the five controls, possibly reflecting a low prevalence of smoking in the study population.

Medication for primary prevention was recommended to 18% of cases, 65% of whom had no history of VTE. Almost all recommendations were for aspirin (data not shown). Anticoagulant (such as heparin or warfarin) use after surgery was recommended to 17% of cases, and 11% of cases were encouraged to use an anticoagulant for a longer duration than originally planned. Fewer than five controls received similar recommendations (data not shown).

Discontinuation of estrogen‐containing oral contraceptives or HRT was recommended to, respectively, 15% and 10% of female cases; heparin treatment during pregnancy was recommended to 5% of cases. Zero controls received these recommendations.

Repeat genetic testing in a clinical lab, or testing for other clotting disorders, was recommended to, respectively, 21% and 13% of cases. Testing in relatives was recommended to 15% of cases. Zero controls received this recommendation.

### 3.5 Behavior and medication changes

Participants answered questions about lifestyle and medication changes prompted by their 23andMe results. Consistent with HCP recommendations, 30% of cases reported beginning to exercise more, 25% took step steps to lose weight, and 12% started to wear compression socks/stockings. In comparison, 12%, 10%, and <2% of controls, respectively, reported making these changes (Table 5). Two percent of cases started taking medication to prevent blood clots, and 4% took a blood thinner such as heparin or warfarin for a longer duration than originally planned. Four and five percent of female cases, respectively, discontinued or changed either an estrogen‐containing contraceptive or HRT. Fewer than 1% of controls made any of these medication changes. A lower percentage of cases (43%) than controls (79%) reported no changes in lifestyle or medication.

We assessed whether receiving thrombophilia results prompted testing of family members of FVL and PT carriers. A higher percentage of cases than controls reported that their family members had had testing for genetic mutations associated with VTE (Table 6), and more cases than controls (28% versus 3%) reported that their relatives received testing because of their genetic results. Among the minority of cases whose relatives received testing because of their results, 48% reported testing through 23andMe; 34%, through a HCP, and 17% through a combination of a HCP and 23andMe.

### 3.6 Perceptions of value and emotional responses to testing

Both cases and controls reported benefit in receiving VTE risk results. Eighty‐one percent of cases and 67% of controls responded that they were satisfied with knowing their genetic risk for VTE; 1% of cases and controls were unsatisfied, and the rest were neither satisfied nor unsatisfied (Table 7A). Similarly, 78% of cases and 58% of controls reported that knowing their genetic probability for VTE had been an advantage, <1% of cases and controls reported that it had been a disadvantage, and the remainder responded “neither an advantage nor disadvantage.” Most participants thought that they could “take steps to reduce the probability of VTE” (87% of cases, 85% of controls), and nearly all (97% of cases, 93% of controls) would choose to learn their genetic risk for VTE again if they could do it all over again (Table 7B).

Approximately half of participants (52% of cases, 56% of controls) reported that they worried the same after learning their genetic risk for VTE; more cases than controls worried more (36% versus 8%), and more controls than cases worried less (36% versus 12%) (Table 7A).

Participants had the opportunity to describe in free text the positive and negative impacts of knowing their genetic risk for VTE (Table 8). Among cases, 642 respondents reported positive impacts, and 201 reported negative impacts; 253 and 47 controls, respectively, reported positive and negative impacts. The most common theme for positive impacts for both cases and controls was ability to take preventative measures. Positive impacts described by cases also included the ability to inform HCPs and to recognize symptoms; those described by controls included increased knowledge of VTE or personal risk, and relief with knowing risk status. The most common theme for negative impacts for both cases and controls was actual or potential for worry. Negative impacts described by cases also included unpleasantness of knowing risk for self and/or family, and those described by controls included potential false sense of security.

## 4 DISCUSSION

Our study assessed outcomes of DTC testing for inherited thrombophilia, including recall of test results, actions taken following receipt of test results, sharing with and recommendations from HCPs, emotional responses, and perception of value. Our data contribute to the discussion of controversial issues in the field, related both to testing for inherited thrombophilia and to DTC testing generally. The Evaluation of Genomic Applications in Practice and Prevention Working Group concluded that there is no evidence that knowledge of *F5*/*F2* status among asymptomatic family members reduced their VTE risk and found that the potential harms may outweigh the benefits (Evaluation of Genomic Applications in Practice and Prevention (EGAPP) Working Group, 2011). Concerns about DTC genetic testing include risk of consumers misinterpreting genetic risk information, potential over‐utilization of medical resources, and negative psychosocial outcomes (Botkin et al., 2010; Leighton & Valverde, 2011; McGuire & Burke, 2008). However, there are indications of consumer and public health interest in greater understanding of thrombophilia. These is also increasing recognition that individuals may derive personal benefit from receiving genetic health risk information, including the ability to understand inherited risk and make healthy choices to control it (Meisel et al., 2015; National Academies of Sciences, Engineering, and Medicine , Mancher, Busta and Downey (U.S.)^2018^). In view of the lack of published evidence in favor of broader testing for *F5*/*F2* (Evaluation of Genomic Applications in Practice and Prevention (EGAPP) Working Group, 2011; Grody et al., 2001; Kujovich, 1993b, 1993c; Stevens et al., 2016), we sought to understand if genetic risk information obtained through DTC testing could have personal utility.

In this study, recall of genetic test results depended on both primary understanding of the VTE report and ability to remember the report content, in most cases, over one year after receiving results, as participants were instructed to rely on their recollection of the test information to answer survey questions. Among participants who had viewed their results, cases were more likely than controls to correctly respond to the question of whether they had at least one risk variant and to correctly perceive their risk as above average (Table 2). These data suggest that receipt of a positive or impactful result is associated with better comprehension and/or recall of the result. Inability to recall a specific *F5* and *F2* genotype was common among cases and controls (Table S3). Together these data indicate that participants could retain the underlying message of having a risk variant or being at higher risk based on genetics, even if they did not recall specific details.

Questions of whether and how DTC genetic testing alters consumer behavior or health are areas of active investigation (Bloss, Wineinger, Darst, Schork, & Topol, 2013; Carere et al., 2014; Hollands et al., 2016; Kaufman, Bollinger, Dvoskin, & Scott, 2012; van der Wouden et al., 2016). In our study, substantial numbers of cases shared their results with HCPs, family, and friends (Table 3). Over half of cases made behavioral or medication changes, the most common of which were exercising more and taking steps to lose weight (Table 5). These interventions are unlikely to induce harm and may improve health generally. The findings are consistent with prior studies showing that people who consider themselves at higher risk for a disease are more likely to discuss results with HCPs and make behavioral changes (Kaufman et al., 2012), but they are inconsistent with those of a large meta‐analysis that found no evidence that communicating DNA‐based risk estimates changes behavior (Hollands et al., 2016). Future investigations might explore the nuances of when and how knowledge of genetic risk, obtained either DTC or in the context of medical care, affects behavior and health outcomes.

Despite not being discussed in the 23andMe VTE report, several cases (12%) reported using compression garments for clot prevention. This may indicate that participants received HCP recommendations or obtained health advice through alternative sources. While compression garments are generally safe, there can be contraindications, particularly in the absence of medical support, and there is insufficient evidence to support their use for primary prophylaxis (Lim & Davies, 2014).

Among cases who discussed their results with HCPs, approximately one third received lifestyle, medication, or additional testing recommendations (Table 4). Unexpectedly, we identified a potential trend of HCPs recommending that asymptomatic cases take aspirin for prevention. An outstanding question is whether HCP recommendations were based on thrombophilia risk—which is not part of clinical management guidelines—or other risks. Given the potential for harm with unnecessary aspirin treatment (McNeil et al., 2018), future studies might explore the reasons for and prevalence of HCP recommendations for aspirin, as well as whether these and other recommendations for medication changes are appropriate in light of current management guidelines.

It is noteworthy that about 15% of cases reported that HCPs recommended testing for relatives, even though many clinical guidelines discourage routine testing of carriers’ family members. The finding is consistent with evidence that clinical testing of VTE patients and their relatives for thrombophilia is widespread (Stevens et al., 2016; Varga & Kujovich, 2012). Interestingly, while 40% of cases reported that their relatives had received genetic testing for VTE‐associated variants, a minority of these relatives (28%) had received testing because of the case's 23andMe results, and approximately, half of these (11% of overall respondents) did so through the medical system (Table 6).

More cases than controls expressed satisfaction with knowing their results and felt that knowing was advantageous (Table 7). Similarly, cases placed more value in knowing their results than controls did. Most participants felt that they could take steps to reduce their risk for VTE, and nearly, all would learn their results again if they could do it over. These results are interesting given that 36% of cases reported worrying more about VTE. Our finding that most participants would choose to know their risk, despite potential for increased worry, is consistent with findings from a previous study that assessed patient experience of a positive FVL test result (Hellmann et al., 2003).

A limitation of this study was that we relied on participants’ self‐report and lacked full comorbidity profiles, which might have helped explain HCP recommendations and actions taken. Another limitation was the variability in time between participants’ receiving the report and responding to the survey, which may have affected accuracy of recollections. Finally, the study population might differ from the general population, including those interested in testing for inherited thrombophilia. The 23andMe participant base is more educated and has higher socioeconomic status than the general U.S. population.

## 5 CONCLUSIONS

This study builds on previous investigations into consumer reactions to DTC testing for disease risk (Carere et al., 2014; Francke et al., 2013), in this instance providing insight into outcomes of testing for VTE risk. We observed moderate rates of cases taking action following receipt of test results (e.g., sharing results with a medical provider or making behavioral changes) and high rates of satisfaction with test results. Further, cases reported positive impacts of receiving test results that included ability to take preventative measures, to inform medical providers, and to recognize signs and symptoms, as well as increased knowledge of VTE and personal risk. Taken together, these results suggest that consumers may experience personal benefit from receiving DTC genetic results for VTE risk that is distinct from the potential benefit in receiving clinically actionable information.

## CONFLICT OF INTEREST

SLE, NAF, BSH, CHW, JLM, HMR, and UF are current or former employees of 23andMe, Inc.

## AUTHORS’ CONTRIBUTIONS

SLE, NAF, CHW, JLM, EAV, and UF conceived and designed the experiments. SLE, NAF, CHW, JLM, HRM, EAV, and UF performed the experiments. NF analyzed the data. SE drafted the manuscript with contributions from all authors. All authors read and approved the final manuscript.
