# Supplementary material for: Direct‐to‐consumer genetic testing for factor V Leiden and prothrombin 20210G>A: the consumer experience
Source: Mol Genet Genomic Med. 2020 Sep 16;8(11):e1468. doi: 10.1002/mgg3.1468 (PMC7667316; doi:10.1002/mgg3.1468)
Supplement: Supplementary file 1 [file MGG3-8-e1468-s001.pdf]

Direct-to-consumer genetic testing for factor V Leiden and prothrombin 20210G>A: the consumer experience

Sarah L. Elson, Nicholas A. Furlotte, Bethann S. Hromatka, Catherine H. Wilson, Joanna L. Mountain, Helen M. Rowbotham, Elizabeth A. Varga, Uta Francke

Supplementary materials include:

- Figure S1 (p. 2)
- Tables S1-S3 (pp. 3-5)
- Survey 1 questions (pp. 6-25)
- Survey 2 questions (pp. 26-33)

Suplemental Figure S1. 23andMe venous thromboembolism (VTE) genetic risk report provided to customers prior to November 2013. **A**. The report included an overview of VTE signs, symptoms, and incidence. **B**. Customers were provided with a personal risk estimate that they could compare to the average population risk estimate for their sex and ancestry. **C**. A technical report provided detailed information about the genetic markers that were included in the report and used to compute a customer's personal risk estimate.

A

Overview | Timeline | MD's Perspective | Resources | Technical Report | Community

Venous Thromboembolism

Printable Version

Venous thromboembolism (VTE) encompasses two related conditions. The first, deep vein thrombosis or DVT, is the formation of a blood clot in a vein deep within the body, usually in the legs. The second, pulmonary embolism (PE), occurs if the clot breaks free and travels through the circulatory system to the lungs. DVT always precedes PE. It is estimated that about 250,000 people are hospitalized with venous thromboembolism in the United States each year, but the incidence is probably much higher as many cases go undiagnosed. Pulmonary embolism is potentially life threatening if prompt medical attention is not received. Therefore, recognizing the symptoms of venous thromboembolism and avoiding risk factors is of paramount importance.

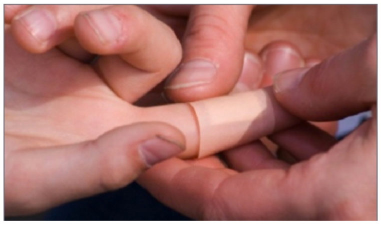

B

Your Results

» Share your health results

Show information for Gus Chandler assuming European ethnicity and an age range of 0-79

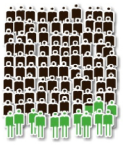

Gus Chandler
12.8 out of 100
men of European ethnicity who share Gus Chandler's genotype will develop Venous Thromboembolism between the ages of 0 and 79.

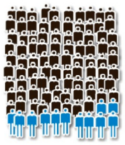

Average
12.3 out of 100
men of European ethnicity will develop Venous Thromboembolism between the ages of 0 and 79.

What does the Odds Calculator show me?

Use the ethnicity and age range selectors above to see the estimated incidence of Venous Thromboembolism due to genetics for men with **Gus Chandler**'s genotype. The 23andMe Odds Calculator assumes that a person is free of the condition at the lower age in the range. You can use the name selector above to see the estimated incidence of Venous Thromboembolism for the genotypes of other people in your account.

The 23andMe Odds Calculator only takes into account effects of markers with known associations that are also on our genotyping chip. Keep in mind that aside from genetics, environment and lifestyle may also contribute to one's risk for Venous Thromboembolism.

C

Technical Report

Gene or region: F5
SNP: rs6025

	SNP used	Genotype	Adjusted Odds Ratio*
Gus Chandler	rs6025	CC	European: 0.96
Nancy Barry	rs6025	CT	European: 4.67
Charlies Foxtrot	rs6025	*NG (not genotyped)*	*NG (not genotyped)*

** Odds ratios are reported for all available ethnicities.*

Factor V is the last clotting factor in the pathway before the activation step that turns prothrombin into thrombin. Clotting is usually kept from spiraling out of control by a feedback loop, similar to the way a thermostat operates. Once enough thrombin has been activated, it binds to a protein called "protein C." Protein C then inactivates factor V, thus cutting off activation of prothrombin into thrombin.

The SNP in the F5 gene causes a change in the protein sequence of factor V that prevents protein C from inactivating it. Since this version of factor V can still participate in the activation of thrombin, a situation results in which thrombin can be turned on but cannot be turned off. Once the clotting cascade is set off (whether appropriately or not), the riskier version of the SNP makes it more difficult to shut it off.

The riskiness of the T version of this SNP is further increased for women who also take hormonal birth control.

(The riskier version of this gene is also sometimes called Factor V Leiden, after the city in the Netherlands where this SNP and its effects on factor V's role in clotting were first discovered.)

The studies whose data we report as applicable to those of "European" ancestry confirmed the association between this SNP and VTE in samples from the Netherlands, Sweden, the United Kingdom, Brazil, Italy, and France.

African and Asian populations appear to have only one version of the SNP, meaning that association studies are very difficult to perform.

Table S1. Survey response rates by sex and number of mutations

	1 mutation		2 or 3 mutations		controls	
	**female**	**male**	**female**	**male**	**female**	**male**
Survey 1 contacted (N)	3818	4416	143	159	5273	6080
Survey 1 responded (N)	655	540	25	24	581	529
Survey 1 response rate (%)	17.2%	12.2%	17.5%	15.1%	11.0%	8.7%
Survey 2 contacted (N)	592	450	25	20	495	434
Survey 2 responded (N)	386	331	18	16	316	258
Survey 2 response rate (%)	65.2%	73.6%	72.0%	80.0%	63.8%	59.4%

Table S2. Personal and family history of VTE

Have you ever been diagnosed with VTE, either deep vein thrombosis (DVT - a blood clot in a deep vein) or pulmonary embolism (PE - a blood clot in the lungs)? (Note: a VTE blood clot is not the same as an arterial blood clot, which may lead to heart attack or stroke.)

	Cases				Controls			
	female (n=640)		male (n=511)		female (n=523)		male (n=475)	
Yes	53	8.3%	45	8.8%	15	2.9%	13	2.7%
No	577	90.2%	455	89.0%	500	95.6%	450	94.7%
I'm not sure	10	1.6%	11	2.2%	8	1.5%	12	2.5%

Have any of your first-degree biological relatives (parent, sibling, child) been diagnosed with a VTE blood clot?

	Cases (n=1231)		Controls (n=1074)	
Yes	286	23.2%	143	13.3%
No	730	59.3%	718	66.9%
I'm not sure	215	17.5%	213	19.8%

[*To participants who reported prior knowledge of genetic risk for VTE*] What was the reason for previous testing? Please check all that apply.

	Cases (n=116)		Controls (n=10)	
I had a deep vein thrombosis (DVT - a blood clot in a deep vein) OR I had a pulmonary embolism (PE - a blood clot in the lungs)	56	48.3%	4	40.0%
Someone in my biological family had a deep vein thrombosis (DVT - a blood clot in a deep vein) OR Someone in my biological family had a pulmonary embolism (PE - a blood clot in the lungs)	57	49.1%	7	70.0%
Someone in my biological family has the F5 and/or F2 mutation	34	29.3%	3	30.0%
Other	27	23.3%	2	20.0%

Table S3. Recall of VTE risk report information.

According to your 23andMe report, what is your chance, given your genetics, of developing a VTE during your lifetime? Note: Please do not refer to your 23andMe report when answering this question, or any of the following questions in this survey.

	1 mutation (n=932)		2 mutations (n=42)		control (n=494)	
Less than 10%	62	6.7%	1	2.4%	197	39.9%
Between 10% and 40%	510	54.7%	7	16.7%	184	37.2%
Greater than 40%	189	20.3%	29	69.0%	25	5.1%
I'm not sure	171	18.3%	5	11.9%	88	17.8%

What are the results of your genetic testing for genes associated with VTE? [*Answer choices included specific F5 and F2 genotypes.*]

Genotype	Participants w/given genotype who provided correct answer choice		Participants w/given genotype who provided incorrect answer choice		Participants w/given genotype who were unsure of results.	
Control	145	30.3%	37	7.7%	296	61.9%
One mutation	265	29.5%	116	12.9%	516	57.5%
Two or more mutations	12	28.6%	7	16.7%	23	54.8%

SURVEY 1

SURVEY TITLE: Venous Thromboembolism
SURVEY DESCRIPTION: The purpose of this project is to collect and update information on health and health behaviors related to the F2 and F5 genes so that we may better understand the relationship between genetic information, behavior, and health.

As part of this project we are interested in understanding your evaluation of 23andMe reports. **Please answer the following questions without referring to your 23andMe reports.**

[1] PAGE: Consent

1.

Consent Document

This consent document provides additional information relevant to the 23andMe Research Initiatives and serves as a supplement to our full consent document, which can be viewed on the 23andMe website.

What is this part of the research about?

In addition to its other studies, 23andMe is conducting targeted research on the impact of compensation on survey data quality. We are recruiting people from outside and/or inside the 23andMe customer base to take part in this research. The purpose of this is to find out how to improve data quality as well as the research participant experience.

How do you participate in the study?

You can take part in this study if you are willing to have your Genetic & Self-Reported Information, such as survey answers, used in research on human health and disease as well as in research on the impact of compensation on survey-taking behavior. There is no cost for participating in this study. Participation in this study is open to adults aged 18 years and older.

What am I agreeing to if I consent to this part of the research?

You have already agreed to let 23andMe researchers use your Genetic & Self-Reported Information for research, as described in the main 23andMe Research consent.

If you additionally agree to this consent document, you agree

- to complete surveys on the 23andMe website regarding health, behavior, ancestry and other IRB-approved research topics, and
- to receive compensation types and value amounts that may differ from those offered to and received by other study participants

What are the additional potential benefits and risks?

Additional benefits: Your participation will allow scientists to study and analyze more thoroughly data on compensation and survey-taking behavior. This may lead to improvements in the research participant experience in future 23andMe studies.

Additional risks: Your participation will mean that you may receive a different type or amount of compensation for research than another participant in the same study. It is possible that you could discover differences in compensation, such as through community forum posts or another family member's participation in the same study. This could potentially lead you to feel unfairly treated, embarrassed, or uncomfortable.

Do I have any alternatives? Can I withdraw from this study?

Your alternative is not to participate in this study. If you are a current 23andMe customer, this will have no effect on your access to your Genetic Information or to the 23andMe Personal Genome Service®. Choosing not to participate in this study will have no effect on your ability to participate in 23andMe Research unrelated to studies of participant compensation (see the main 23andMe consent document for details).

At any time, you may choose to withdraw all or some of your Genetic & Self-Reported information from this 23andMe study by changing your consent status within the Privacy/Consent tab on the 23andMe Settings page.

If you withdraw from the study, you will still be allowed full access to your Genetic Information and to the Personal Genome Service®. 23andMe will prevent your study data from being used in new 23andMe research occurring after 30 days from receipt of your request. Any research on your data that has been performed or published prior to this date will not be reversed, undone, or withdrawn.

Who do I contact if I have questions?

If you have *general questions and need help with 23andMe's service,* please go to the following webpage:

23andMe Customer Care
https://customercare.23andme.com/home

If you *suffer a research-related injury, or if you have a question about subjects' rights,* please contact the following:

23andMe Human Protections Administrator
Email: hpa@23andme.com

If you have *any questions or concerns about research that you do not wish to discuss with 23andMe,*click here to contact the following independent, impartial research review board:

E&I Review Services
Email: subject@eandireview.com
Phone: 1-800-472-3241

Please choose one of the following options:

( ) Yes, I'd like to take part in this study. I am this person, I am at least 18 years old, I have read this document, and I CONSENT to this study.
( ) Yes, I'd like to take part in this study. I am legally authorized to give consent for this person, this person is at least 18 years old, I have read this document, and I CONSENT to this study.
( ) No, thank you. I do not want to participate in this study.

{IF ((#1=Yes, I'd like to take part in this study. I am this person, I am at least 18 years old, I have read this document, and I CONSENT to this study. OR #1=Yes, I'd like to take part in this study. I am legally authorized to give consent for this person, this person is at least 18 years old, I have read this document, and I CONSENT to this study.))}

[2] PAGE: Genetic Results

2. When did you first receive your 23andMe genetic results?
( ) Within the last 3 months
( ) Between 3 months and 1 year ago
( ) Between 1 and 2 years ago
( ) More than 2 years ago
( ) I'm not sure

3. Have you looked at your 23andMe "Venous Thromboembolism (VTE)" report? A VTE is the blocking of a vein by a blood clot.
( ) Yes
( ) No
( ) I'm not sure

{IF ((#3=Yes))}

4. According to your 23andMe report, what is your chance, given your genetics, of developing a VTE during your lifetime?

Note: Please do not refer to your 23andMe report when answering this question, or any of the following questions in this survey.
( ) Less than 10%
( ) Between 10% and 40%
( ) Greater than 40%
( ) I'm not sure

{IF ((sex = None))}

5. What is your sex?
( ) Male
( ) Female
( ) Other

{IF ((#3=Yes) AND (#5=Female))}

6. 23andMe also has a Drug Response Report called "Oral Contraceptives, Hormone Replacement Therapy, and Risk of Venous Thromboembolism", which discusses the association between hormone use

and developing VTE in light of genetic results. What do you understand your result to be?
( ) If I take oral hormone replacement therapy or an estrogen-containing contraceptive (which may be administered through an oral pill, a patch, a ring, or other) my risk of VTE will increase
( ) If I take oral hormone replacement therapy or an estrogen-containing contraceptive (which may be administered through an oral pill, a patch, a ring, or other) my risk of VTE will be unchanged
( ) If I take oral hormone replacement therapy or an estrogen-containing contraceptive (which may be administered through an oral pill, a patch, a ring, or other) my risk of VTE will decrease
( ) I don't know

{IF ((#3=Yes))}

7. According to your 23andMe report, which of the following increases an individual's risk of developing VTE? Please check all that apply.
[ ] Hormone use
[ ] Eating a high protein diet
[ ] Smoking
[ ] Television viewing
[ ] Obesity
[ ] Consuming red wine
[ ] Immobilization or sitting still for long periods of time
[ ] Cell phone use
[ ] I'm not sure

{IF ((#3=Yes))}

8. What are the results of your genetic testing for genes associated with VTE?
( ) I have one mutated copy of F5 (Factor V Leiden)
( ) I have two mutated copies of F5 (Factor V Leiden)
( ) I have one mutated copy of F2 (prothrombin or factor II)
( ) I have two mutated copies of F2 (prothrombin or factor II)
( ) I have mutated copies of both F5 and F2 (Factor V Leiden and prothrombin or factor II)
( ) I don't have either of these mutations
( ) I'm not sure

9. Did you already know about your genetic risk for VTE before you received your 23andMe results?
( ) Yes, I was tested for these mutations before I received my 23andMe results
( ) No

{IF ((#9=Yes, I was tested for these mutations before I received my 23andMe results))}

10. What was the reason for previous testing? Please check all that apply.
[ ] I had a deep vein thrombosis (DVT - a blood clot in a deep vein)
[ ] I had a pulmonary embolism (PE - a blood clot in the lungs)
[ ] Someone in my biological family had a deep vein thrombosis (DVT - a blood clot in a deep vein)
[ ] Someone in my biological family had a pulmonary embolism (PE - a blood clot in the lungs)
[ ] Someone in my biological family has the F5 and/or F2 mutation
[ ] Other:_____________

{IF ((#3=No OR #3=I'm not sure))}

11. What is your chance, given your genetics, of developing a VTE during your lifetime?

Note: Please do not refer to your 23andMe report when answering this question, or any of the following questions in this survey.
( ) Less than 10%
( ) Between 10% and 40%
( ) Greater than 40%
( ) I'm not sure

{IF ((#3=No OR #3=I'm not sure) AND (#5=Female))}

12. We are interested in the association between hormone use and developing VTE in light of genetic results. What do you understand your result to be?
( ) If I take oral hormone replacement therapy or an estrogen-containing contraceptive (which may be administered through an oral pill, a patch, a ring, or other) my risk of VTE will increase
( ) If I take oral hormone replacement therapy or an estrogen-containing contraceptive (which may be administered through an oral pill, a patch, a ring, or other) my risk of VTE will be unchanged
( ) If I take oral hormone replacement therapy or an estrogen-containing contraceptive (which may be administered through an oral pill, a patch, a ring, or other) my risk of VTE will decrease
( ) I don't know

{IF ((#3=No OR #3=I'm not sure))}

13. Which of the following increases an individual's risk of developing VTE? Please check all that apply.
[ ] Hormone use
[ ] Eating a high protein diet
[ ] Smoking
[ ] Television viewing
[ ] Obesity
[ ] Consuming red wine
[ ] Immobilization or sitting still for long periods of time
[ ] Cell phone use
[ ] I'm not sure

{IF ((#3=No OR #3=I'm not sure))}

14. What are the results of your genetic testing for genes associated with VTE?
( ) I have one mutated copy of F5 (Factor V Leiden)
( ) I have two mutated copies of F5 (Factor V Leiden)
( ) I have one mutated copy of F2 (prothrombin or factor II)
( ) I have two mutated copies of F2 (prothrombin or factor II)
( ) I have mutated copies of both F5 and F2 (Factor V Leiden and prothrombin or factor II)
( ) I don't have either of these mutations
( ) I'm not sure

15. To your knowledge, does 23andMe test for all genetic changes increasing the risk of VTE?
( ) Yes
( ) No
( ) I'm not sure

16. If someone has one copy of the F5 or F2 mutation, which of their family members have an approximately 50% chance of also carrying it? Please check all that apply.
[ ] Mother
[ ] Father
[ ] Sister
[ ] Brother
[ ] Half-sibling
[ ] Child

17. Are females more likely to inherit F5 and F2 mutations than males?
( ) Yes
( ) No
( ) I'm not sure

{IF ((#1=Yes, I'd like to take part in this study. I am this person, I am at least 18 years old, I have read this document, and I CONSENT to this study. OR #1=Yes, I'd like to take part in this study. I am legally authorized to give consent for this person, this person is at least 18 years old, I have read this document, and I CONSENT to this study.))}

[3] PAGE: Personal and Family Medical History

18. Would you say that in general your health is:
( ) Excellent
( ) Very good
( ) Good
( ) Fair
( ) Poor

19. Have you ever been diagnosed with VTE, either deep vein thrombosis (DVT - a blood clot in a deep vein) or pulmonary embolism (PE - a blood clot in the lungs)?

(Note: a VTE blood clot is not the same as an arterial blood clot, which may lead to heart attack or stroke.)
( ) Yes, I have been diagnosed with VTE
( ) No, I have not been diagnosed with VTE
( ) I'm not sure

{IF ((#19=Yes, I have been diagnosed with VTE))}

20. Were you diagnosed with VTE before you received your 23andMe results, after you received your 23andMe results, or both before and after you received your 23andMe results?
( ) Before I received my 23andMe results
( ) After I received my 23andMe results
( ) Both before and after I received my 23andMe results
( ) I'm not sure

{IF ((#19=Yes, I have been diagnosed with VTE))}

21. How many years old were you when you had your first VTE diagnosis?

{IF ((#19=Yes, I have been diagnosed with VTE))}

22. Where in the body did your first VTE occur?
( ) Arm
( ) Leg
( ) Lung
( ) Elsewhere:_____________

{IF ((#19=Yes, I have been diagnosed with VTE))}

23. In the 6 weeks before your first VTE, did you have or experience any of the following? Please check all that apply.
[ ] Major surgery (requiring general anesthesia and lasting at least 30 minutes)
[ ] Hospitalization or other prolonged immobility
[ ] Physical trauma or injury
[ ] Estrogen-containing contraceptive
[ ] Long-distance travel (sitting in place for at least 8 hours, such as on a plane, bus or train)
[ ] Pregnancy or childbirth
[ ] Oral hormone replacement therapy
[ ] Cancer
[ ] Treatment for cancer
[ ] Central venous catheter
[ ] None of the above
[ ] I'm not sure

{IF ((#19=Yes, I have been diagnosed with VTE) AND (#20=Both before and after I received my 23andMe results OR #20=Before I received my 23andMe results))}

24. Were you diagnosed with more than one incidence of VTE before you received your 23andMe results?
( ) Yes
( ) No

{IF ((#19=Yes, I have been diagnosed with VTE) AND (#20=Both before and after I received my 23andMe results OR #20=Before I received my 23andMe results) AND (#24=Yes))}

25. In total, how many incidences of VTE were you diagnosed with before you received your 23andMe results?

{IF ((#19=Yes, I have been diagnosed with VTE) AND (#20=After I received my 23andMe results OR #20=Both before and after I received my 23andMe results))}

26. Were you diagnosed with more than one incidence of VTE after you received your 23andMe results?
( ) Yes
( ) No

{IF ((#19=Yes, I have been diagnosed with VTE) AND (#20=After I received my 23andMe results OR #20=Both before and after I received my 23andMe results) AND (#26=Yes))}

27. In total, how many incidences of VTE were you diagnosed with after you received your 23andMe

results?

28. Are you currently taking any oral or injectable blood thinners (anticoagulants) such as warfarin (Coumadin) or heparin?
( ) Yes
( ) No

{IF ((#19=No, I have not been diagnosed with VTE OR #19=I'm not sure))}

29. Have you ever had or experienced any of the following? Please check all that apply.
[ ] Major surgery (requiring general anesthesia and lasting at least 30 minutes)
[ ] Hospitalization or other prolonged immobility
[ ] Physical trauma or injury
[ ] Estrogen-containing contraceptive
[ ] Long-distance travel (sitting in place for at least 8 hours, such as on a plane, bus or train)
[ ] Pregnancy or childbirth
[ ] Oral hormone replacement therapy
[ ] Cancer
[ ] Treatment for cancer
[ ] Central venous catheter
[ ] None of the above
[ ] I'm not sure

{IF ((#19=No, I have not been diagnosed with VTE OR #19=I'm not sure) AND (#29=Major surgery (requiring general anesthesia and lasting at least 30 minutes)))}

30. Please tell us about when each of your major surgeries (requiring general anesthesia and lasting at least 30 minutes) occurred by checking all that apply.
[ ] In the last 6 weeks
[ ] 6 weeks to 3 months ago
[ ] 3 months to 6 months ago
[ ] 6 months to 1 year ago
[ ] More than 1 year ago
[ ] I'm not sure

{IF ((#19=No, I have not been diagnosed with VTE OR #19=I'm not sure) AND (#29=Hospitalization or other prolonged immobility))}

31. Please tell us about when each of your hospitalizations or other prolonged immobilities occurred by checking all that apply.
[ ] In the last 6 weeks
[ ] 6 weeks to 3 months ago
[ ] 3 months to 6 months ago
[ ] 6 months to 1 year ago
[ ] More than 1 year ago
[ ] I'm not sure

{IF ((#19=No, I have not been diagnosed with VTE OR #19=I'm not sure) AND (#29=Physical trauma or injury))}

32. Please tell us about when each of your physical traumas or injuries occurred by checking all that apply.
[ ] In the last 6 weeks
[ ] 6 weeks to 3 months ago
[ ] 3 months to 6 months ago
[ ] 6 months to 1 year ago
[ ] More than 1 year ago
[ ] I'm not sure

{IF ((#19=No, I have not been diagnosed with VTE OR #19=I'm not sure) AND (#29=Estrogen-containing contraceptive) AND (#5=Female))}

33. Please tell us about when each of your uses of an estrogen-containing contraceptive occurred by checking all that apply.
[ ] In the last 6 weeks
[ ] 6 weeks to 3 months ago
[ ] 3 months to 6 months ago
[ ] 6 months to 1 year ago
[ ] More than 1 year ago
[ ] I'm not sure

{IF ((#19=No, I have not been diagnosed with VTE OR #19=I'm not sure) AND (#29=Long-distance travel (sitting in place for at least 8 hours, such as on a plane, bus or train)))}

34. Please tell us about when each of your long-distance travels (sitting in place for at least 8 hours, such as on a plane, bus or train) occurred by checking all that apply.
[ ] In the last 6 weeks
[ ] 6 weeks to 3 months ago
[ ] 3 months to 6 months ago
[ ] 6 months to 1 year ago
[ ] More than 1 year ago
[ ] I'm not sure

{IF ((#19=No, I have not been diagnosed with VTE OR #19=I'm not sure) AND (#29=Pregnancy or childbirth) AND (#5=Female))}

35. Please tell us about when each of your pregnancies or childbirths occurred by checking all that apply.
[ ] In the last 6 weeks
[ ] 6 weeks to 3 months ago
[ ] 3 months to 6 months ago
[ ] 6 months to 1 year ago
[ ] More than 1 year ago
[ ] I'm not sure

{IF ((#19=No, I have not been diagnosed with VTE OR #19=I'm not sure) AND (#29=Oral hormone replacement therapy))}

36. Please tell us about when each of your uses of oral hormone replacement therapy occurred by checking all that apply.
[ ] In the last 6 weeks

[ ] 6 weeks to 3 months ago
[ ] 3 months to 6 months ago
[ ] 6 months to 1 year ago
[ ] More than 1 year ago
[ ] I'm not sure

{IF ((#19=No, I have not been diagnosed with VTE OR #19=I'm not sure) AND (#29=Cancer))}

37. Please tell us about when each of your cancers occurred by checking all that apply.
[ ] In the last 6 weeks
[ ] 6 weeks to 3 months ago
[ ] 3 months to 6 months ago
[ ] 6 months to 1 year ago
[ ] More than 1 year ago
[ ] I'm not sure

{IF ((#19=No, I have not been diagnosed with VTE OR #19=I'm not sure) AND (#29=Treatment for cancer))}

38. Please tell us about when each of your treatments for cancer occurred by checking all that apply.
[ ] In the last 6 weeks
[ ] 6 weeks to 3 months ago
[ ] 3 months to 6 months ago
[ ] 6 months to 1 year ago
[ ] More than 1 year ago
[ ] I'm not sure

{IF ((#19=No, I have not been diagnosed with VTE OR #19=I'm not sure) AND (#29=Central venous catheter))}

39. Please tell us about when you had each of your central venous catheters in by checking all that apply.
[ ] In the last 6 weeks
[ ] 6 weeks to 3 months ago
[ ] 3 months to 6 months ago
[ ] 6 months to 1 year ago
[ ] More than 1 year ago
[ ] I'm not sure

40. Have any of your first-degree biological relatives (parent, sibling, child) been diagnosed with a VTE blood clot?
( ) Yes
( ) No
( ) I'm not sure

{IF ((#40=Yes))}

41. Which of your biological relatives have been diagnosed with VTE? Please check all that apply.
[ ] Mother
[ ] Father

[ ] Sister
[ ] Brother
[ ] Children
[ ] Aunt
[ ] Uncle
[ ] Cousin
[ ] Grandparent
[ ] Other:_____________
[ ] None of the above

{IF ((weight_g = None OR days_since_health_intake_completed > 182))}

42. What is your current weight?

{IF ((chronic_kidney_disease = None))}

43. Have you ever been diagnosed with chronic kidney disease?
( ) Yes
( ) No
( ) I'm not sure

{IF ((days_smoke = None OR days_since_tobacco_completed > 182))}

44. Do you typically smoke cigarettes every day, some days, or not at all?
( ) Every day
( ) Some days
( ) Not at all

{IF ((#1=Yes, I'd like to take part in this study. I am this person, I am at least 18 years old, I have read this document, and I CONSENT to this study. OR #1=Yes, I'd like to take part in this study. I am legally authorized to give consent for this person, this person is at least 18 years old, I have read this document, and I CONSENT to this study.))}

[4] PAGE: Results and Follow-Up

45. Since you received your 23andMe results, with whom have you discussed your genetic risk for VTE? Please check all that apply.
[ ] Spouse or significant other
[ ] Mother
[ ] Father
[ ] Sister(s)
[ ] Brother(s)
[ ] Child(ren)
[ ] Aunt(s)
[ ] Uncle(s)
[ ] Cousin(s)
[ ] Grandparent(s)
[ ] Friend(s)
[ ] Health care provider(s)
[ ] Online forum (23andMe or other)

[ ] None of the above

{IF ((#45=Health care provider(s)))}

46. With which health care providers have you discussed your genetic risk for VTE? Please check all that apply.
[ ] Primary care physician
[ ] Hematologist
[ ] Surgeon
[ ] Obstetrician/gynecologist
[ ] Medical geneticist
[ ] Genetic counselor
[ ] Nurse practitioner
[ ] Pharmacist
[ ] Physician assistant
[ ] Nurse
[ ] Other:_____________
[ ] None of the above

{IF ((#45=Health care provider(s)))}

47. When you shared your 23andMe results with your healthcare provider(s), did he/she recommend any changes to your lifestyle, any changes to your medications, or additional testing?
( ) Yes
( ) No

{IF ((#45=Health care provider(s)) AND (#47=Yes))}

48. What did your health care provider recommend? Please check all that apply.
[ ] Lose weight
[ ] Stop smoking
[ ] Wear compression socks/stockings
[ ] Exercise more
[ ] Discontinue or change estrogen-containing contraceptive
[ ] Discontinue or change hormone replacement therapy
[ ] Take medication to prevent blood clots
[ ] Use heparin during pregnancy
[ ] Use a blood thinner (anticoagulant) such as heparin or warfarin after surgery
[ ] Use a blood thinner (anticoagulant) such as heparin or warfarin for a longer duration
[ ] Repeat F5 and F2 testing in a clinical laboratory
[ ] Have testing for other clotting disorders
[ ] Suggest relatives have testing for clotting disorders
[ ] Other:_____________
[ ] None of the above

{IF ((#45=Health care provider(s)) AND (#47=Yes) AND (#48=Take medication to prevent blood clots))}

49. What medication did your health care provider recommend that you take to prevent blood clots?

{IF ((#5=Female) AND (#45!=Health care provider(s)))}

50. Under what circumstances would you want to talk to a healthcare provider about your genetic risk for VTE? Please check all that apply.
[ ] If I were considering using an estrogen-containing contraceptive
[ ] If I were considering hormone-replacement therapy
[ ] If I were considering pregnancy or became pregnant
[ ] Before major surgery (requiring general anesthesia and lasting at least 30 minutes)
[ ] In the event of physical trauma or injury
[ ] In the event of hospitalization or other prolonged immobility
[ ] If I were diagnosed with cancer
[ ] If my daughter was considering using an estrogen-containing contraceptive
[ ] If my daughter was considering hormone-replacement therapy
[ ] If my daughter was considering pregnancy
[ ] Other:_____________
[ ] None of the above
[ ] I'm not sure

{IF ((#5=Male) AND (#45!=Health care provider(s)))}

51. Under what circumstances would you want to talk to a healthcare provider about your genetic risk for blood clots? Please check all that apply.
[ ] Before major surgery (requiring general anesthesia and lasting at least 30 minutes)
[ ] In the event of physical trauma or injury
[ ] In the event of hospitalization or other prolonged immobility
[ ] If I were diagnosed with cancer
[ ] If my daughter was considering using an estrogen-containing contraceptive
[ ] If my daughter was considering hormone-replacement therapy
[ ] If my daughter was considering pregnancy
[ ] Other:_____________
[ ] None of the above
[ ] I'm not sure

{IF ((#3=Yes))}

52. Have you made any of the following changes as a result of your 23andMe report on genetic risk for VTE? Please check all that apply.
[ ] Stopped smoking
[ ] Took steps to lose weight
[ ] Started wearing compression socks/stockings
[ ] Exercise more
[ ] Discontinued or changed estrogen-containing contraceptive
[ ] Discontinued or changed hormone replacement therapy
[ ] Started taking medication to prevent blood clots
[ ] Took a blood thinner (anticoagulant) such as heparin during pregnancy
[ ] Took a blood thinner (anticoagulant) such as heparin or warfarin after surgery
[ ] Took a blood thinner (anticoagulant) such as heparin or warfarin for a longer duration than originally planned
[ ] Other:_____________
[ ] Made no changes

{IF ((#3=Yes) AND (#5=Female))}

53. Did you ask your physician about receiving a blood thinner (anticoagulant) such as heparin during pregnancy because of your 23andMe report on genetic risk for VTE?
( ) Yes
( ) No
( ) I haven't been pregnant since finding out my result
( ) I'm not sure

{IF ((#3=Yes))}

54. Did you ask your physician about receiving a blood thinner (anticoagulant) such as heparin or warfarin after surgery as a result of your 23andMe report on genetic risk for VTE?
( ) Yes
( ) No
( ) I haven't had a surgery since finding out my 23andMe result
( ) I'm not sure

55. Which of your biological relatives has had genetic testing? Please check all that apply.
[ ] Spouse or significant other
[ ] Mother
[ ] Father
[ ] Sister(s)
[ ] Brother(s)
[ ] Child(ren)
[ ] Aunt(s)
[ ] Uncle(s)
[ ] Cousin(s)
[ ] Grandparent(s)
[ ] Other:_____________
[ ] None of the above

{IF ((#55=Spouse or significant other OR #55=Mother OR #55=Father OR #55=Sister(s) OR #55=Brother(s) OR #55=Child(ren) OR #55=Aunt(s) OR #55=Uncle(s) OR #55=Cousin(s) OR #55=Grandparent(s) OR #55=Other:))}

56. Did any of your relatives get genetic testing because of your results?
( ) Yes
( ) No
( ) I'm not sure

{IF ((#56=Yes))}
Which of your biological relatives has had genetic testing because of your results? Please check the choice that applies for each case.

{IF ((#55=Spouse or significant other))}
57. Spouse or significant other

( )Tested because of my result   ( )Not tested because of my result   ( )I'm not sure

{IF ((#55=Mother))}
58. Mother

( )Tested because of my result ( )Not tested because of my result ( )I'm not sure

{IF ((#55=Father))}
59. Father

( )Tested because of my result ( )Not tested because of my result ( )I'm not sure

{IF ((#55=Sister(s)))}
60. Sister(s)

( )Tested because of my result ( )Not tested because of my result ( )I'm not sure

{IF ((#55=Brother(s)))}
61. Brother(s)

( )Tested because of my result ( )Not tested because of my result ( )I'm not sure

{IF ((#55=Child(ren)))}
62. Child(ren)

( )Tested because of my result ( )Not tested because of my result ( )I'm not sure

{IF ((#55=Aunt(s)))}
63. Aunt(s)

( )Tested because of my result ( )Not tested because of my result ( )I'm not sure

{IF ((#55=Uncle(s)))}
64. Uncle(s)

( )Tested because of my result ( )Not tested because of my result ( )I'm not sure

{IF ((#55=Cousin(s)))}
65. Cousin(s)

( )Tested because of my result ( )Not tested because of my result ( )I'm not sure

{IF ((#55=Grandparent(s)))}
66. Grandparent(s)

( )Tested because of my result ( )Not tested because of my result ( )I'm not sure

{IF ((#55=Other:))}
67. Other

( )Tested because of my result ( )Not tested because of my result ( )I'm not sure

Did your biological relatives who were tested receive a positive or negative result for the F5 and/or F2 mutations? Please check the choice that applies for each case.

{IF ((#55=Spouse or significant other))}
68. Spouse or significant other

( )Positive ( )Negative ( )I'm not sure

{IF ((#55=Mother))}
69. Mother

( )Positive ( )Negative ( )I'm not sure

{IF ((#55=Father))}
70. Father

( )Positive ( )Negative ( )I'm not sure

{IF ((#55=Sister(s)))}
71. Sister(s)

( )Positive ( )Negative ( )I'm not sure

{IF ((#55=Brother(s)))}
72. Brother(s)

( )Positive ( )Negative ( )I'm not sure

{IF ((#55=Child(ren)))}
73. Child(ren)

( )Positive ( )Negative ( )I'm not sure

{IF ((#55=Aunt(s)))}
74. Aunt(s)

( )Positive ( )Negative ( )I'm not sure

{IF ((#55=Uncle(s)))}
75. Uncle(s)

( )Positive ( )Negative ( )I'm not sure

{IF ((#55=Cousin(s)))}
76. Cousin(s)

( )Positive ( )Negative ( )I'm not sure

{IF ((#55=Grandparent(s)))}
77. Grandparent(s)

( )Positive ( )Negative ( )I'm not sure

{IF ((#55=Other:))}
78. Other

( )Positive ( )Negative ( )I'm not sure

{IF ((#55!=None of the above) AND ())}

79. Where were your relatives genetically tested?
( ) 23andMe
( ) Through a health care provider
( ) A combination of 23andMe and health care providers
( ) I'm not sure

{IF ((#1=Yes, I'd like to take part in this study. I am this person, I am at least 18 years old, I have read this document, and I CONSENT to this study. OR #1=Yes, I'd like to take part in this study. I am legally authorized to give consent for this person, this person is at least 18 years old, I have read this document, and I CONSENT to this study.))}

[5] PAGE: Awareness of Signs, Symptoms, and Risk Factors for VTE

80. What are the characteristic signs and symptoms of deep vein thrombosis (a blood clot in a vein)? Please check all that apply.
[ ] Pain in both legs
[ ] Pain in one leg with swelling

[ ] Coughing up blood
[ ] Redness and warmth of one leg
[ ] Pain and swelling of both arms
[ ] Bruising of the leg
[ ] I'm not sure

81. What are the characteristic signs and symptoms of pulmonary embolism (a blood clot in the lungs)? Please check all that apply.
[ ] Chest pain or discomfort
[ ] Shortness of breath
[ ] Lightheadedness
[ ] Coughing up blood
[ ] Excessive sweating
[ ] Sneezing
[ ] Rapid heartbeat
[ ] Wheezing
[ ] Sense of anxiety
[ ] Forgetfulness
[ ] I'm not sure

{IF ((#3=Yes) AND (#1=Yes, I'd like to take part in this study. I am this person, I am at least 18 years old, I have read this document, and I CONSENT to this study. OR #1=Yes, I'd like to take part in this study. I am legally authorized to give consent for this person, this person is at least 18 years old, I have read this document, and I CONSENT to this study.))}

[6] PAGE: Attitudes Toward Genetic Knowledge

82. Are you satisfied or unsatisfied that you know your genetic probability for VTE?
( ) Satisfied
( ) Neither satisfied nor unsatisfied
( ) Unsatisfied

83. How important is it to you to know your genetic probability for VTE?
( ) Very important
( ) Somewhat important
( ) Moderately important
( ) Slightly important
( ) Not at all important

84. Do you think that you can take steps to reduce the probability of VTE?
( ) Yes
( ) No
( ) I'm not sure

85. Has knowing your genetic probability for VTE been an advantage or disadvantage for you?
( ) Advantage
( ) Neither an advantage nor disadvantage
( ) Disadvantage

86. Does knowing your genetic probability for VTE make you worry more or worry less about developing

VTE?
( ) Worry more
( ) Worry the same
( ) Worry less

87. Do you think that others should be tested for their genetic risk for VTE?
( ) Yes
( ) No
( ) I'm not sure

88. Please describe any positive impact of knowing your genetic risk for VTE.

89. Please describe any negative impact of knowing your genetic risk for VTE.

90. Please describe anything about your experience with VTE and genetic risk for VTE that you think is important but has not been reflected in the previous questions.

91. Do you give 23andMe permission to quote your responses to the open-ended questions in this survey without using your name?
( ) Yes
( ) No
When you signed up for 23andMe, how interested were you in the following?
92. Ancestry

( )Not at all interested ( )Somewhat interested ( )Very interested

93. Genetic-based risk of disease or health conditions

( )Not at all interested ( )Somewhat interested ( )Very interested

94. Genetic-based response to medications

( )Not at all interested ( )Somewhat interested ( )Very interested

95. Carrier status for inherited conditions

( )Not at all interested ( )Somewhat interested ( )Very interested

{IF ((#1=Yes, I'd like to take part in this study. I am this person, I am at least 18 years old, I have read this document, and I CONSENT to this study. OR #1=Yes, I'd like to take part in this study. I am legally authorized to give consent for this person, this person is at least 18 years old, I have read this document, and I CONSENT to this study.))}

[7] PAGE: Demographics

96. What is the highest level of education you completed?
( ) Less than first grade
( ) First to eighth grade
( ) Some high school, no diploma

( ) High school diploma or equivalent (for example: GED)
( ) Some college but no degree
( ) Associate college degree
( ) Bachelor's college degree (for example: BA, AB, BS)
( ) Master's degree (for example: MA, MS, MEng, Med, MSW, MBA)
( ) Professional school degree (for example: MD, DDS, DVM, LLB, JD)
( ) Doctorate degree (for example: PhD, EdD)
( ) Other:_____________

97. What is your household annual income?
( ) $14,999 or less
( ) $15,000 to $24,999
( ) $25,000 to $39,999
( ) $40,000 to $59,999
( ) $60,000 to $89,999
( ) $90,000 to $124,999
( ) $125,000 or more
( ) I'd rather not say

{IF ((ancestry_ethnicity_hh = None))}

98. What best describes your ancestry/ethnicity? Please check all that apply.
[ ] European or White
[ ] African American or Black
[ ] Latino or Hispanic
[ ] Asian (e.g. Chinese, Indian, Kazakh, Thai)
[ ] Middle Eastern or North African
[ ] Sub-Saharan African
[ ] Native American or Alaska Native
[ ] Pacific Islander or Oceanian
[ ] Other
[ ] I'm not sure

SURVEY 2

This survey is designed to find out more about your health and your attitudes and understanding of the causes and consequences of Venous Thromboembolism (VTE). VTE is a condition in which blood clots form in veins (deep vein thrombosis, DVT), and occasionally travel through the bloodstream to the lungs (pulmonary embolism, PE). This survey is a followup to our VTE Baseline survey which you completed approximately 9 months ago in 2015 between March 19th and May 1st.

PAGE: Basic VTE Information

In the last 9 months, have you looked at your 23andMe report on Venous Thromboembolism (VTE)?

( ) Yes
( ) No
( ) I'm not sure

Compared to others of the same sex and ethnicity, what is your chance of developing VTE during your lifetime?

Note: Please do not refer to your 23andMe report when answering this question, or any of the following questions in this survey.

( ) Lower than average ( ) Average
( ) Higher than average ( ) I'm not sure

What are the results of your genetic testing for genes associated with VTE? ( ) I have one or more mutations that increase the risk of VTE
( ) I do not have any mutations that increase the risk of VTE
( ) I'm not sure

[If above=I have one or more mutations...]
Did you have any subsequent genetic testing to confirm your initial VTErelated mutations result?

( ) Yes ( ) No

PAGE: Personal and Family Medical History

In the last 9 months (since taking our VTE baseline survey) have you been diagnosed with VTE, either deep vein thrombosis (DVT, a blood clot in a deep vein) and/or pulmonary embolism (PE, a blood clot in the lungs)?

(Note: a DVT / PE blood clot is not the same as an arterial blood clot, which may lead to heart attack or stroke.)

( ) Yes, in the last 9 months I have been diagnosed with VTE
( ) No, in the last 9 months I have not been diagnosed with VTE ( ) I'm not sure

{IF (above=Yes, I have been diagnosed with VTE))}

Where in the body have you had VTE? Please check all that apply. ( ) Arm

( ) Leg
( ) Lung
( ) Elsewhere:_____________

In the last 9 months did you have or experience any of the following? Please check all that apply.

[ ] Major surgery (requiring general anesthesia and lasting at least 30 minutes) [ ] Hospitalization or other prolonged immobility
[ ] Physical trauma or injury
[ ] Estrogencontaining contraceptive

[ ] Longdistance travel (sitting in place for at least 4 hours) [ ] Pregnancy or childbirth
[ ] Oral hormone replacement therapy
[ ] Cancer

[ ] Treatment for cancer
[ ] Central venous catheter [ ] None of the above
[ ] I'm not sure

[If above=Major surgery and VTE diagnosis=yes]
Was your surgery in the last 9 months performed after you were diagnosed with VTE to treat your VTE?

Yes No

[If above=Hospitalization and VTE diagnosis=yes]
Did your hospitalization in the last 9 months occur after you were diagnosed with VTE to treat your VTE?

Yes No

[If above=Central venous catheter and VTE diagnosis=yes]

Was your central venous catheter inserted in the last 9 months after you were diagnosed with VTE to treat your VTE?

Yes No

[If above=Major surgery]

In the last 9 months, which of the following surgical procedures did you have? Please check all that apply.

Total hip replacement
Hip fracture surgery
Total knee replacement
Lower leg fracture/injury with immobilization Spinal surgery
Major abdominal or cardiac surgery
Other (text fill in)

[If above=Major surgery or Hospitalization]
Following your surgery / hospitalization in the last 9 months, did you receive medication to lower the risk of abnormal blood clotting (for example, aspirin, heparin or warfarin)?

- Yes
- No
- I’m not sure

[If yes]
What medication did you receive following your surgery / hospitalization in the last 9 months? Please check all that apply.

- Aspirin
- Heparin
- Warfarin
- I’m not sure
- None of the above

Are you currently taking any oral or injectable blood thinners (anticoagulants) such as warfarin (Coumadin) or heparin?

( ) Yes ( ) No

In the last 9 months, have any of your firstdegree biological relatives (parent, sibling, child) been diagnosed with VTE (DVT or PE)?

( ) Yes
( ) No
( ) I'm not sure

{IF ((above=Yes))}

In the last 9 months, which of your biological relatives have been diagnosed with VTE? Please check all that apply.

[ ] Mother [ ] Father [ ] Sister
[ ] Brother [ ] Child

[ ] Aunt
[ ] Uncle
[ ] Cousin
[ ] Grandparent
[ ] Other:_____________ [ ] None of the above

{IF ((weight_g = None OR days_since_health_intake_completed > 182))} What is your current weight?

[4] PAGE: Results and FollowUp

In the last 9 months, with whom have you discussed your genetic risk for VTE? Please check all that apply.

[ ] Spouse or significant other [ ] Mother
[ ] Father
[ ] Sister

[ ] Brother
[ ] Daughter
[ ] Son
[ ] Aunt
[ ] Uncle
[ ] Cousin
[ ] Grandparent
[ ] Friend
[ ] Healthcare provider
[ ] Online forum (23andMe or other) [ ] None of the above

{IF (above=Health care provider(s)))}

In the last 9 months, with which healthcare providers have you discussed your genetic risk for VTE? Please check all that apply.

[ ] Primary care physician
[ ] Hematologist
[ ] Surgeon
[ ] Obstetrician/gynecologist [ ] Medical geneticist

[ ] Genetic counselor [ ] Nurse practitioner
[ ] Pharmacist
[ ] Physician assistant [ ] Nurse

[ ] Other:_____________ [ ] None of the above

{IF ((above=Health care provider(s)))}

In the last 9 months has your healthcare provider(s), recommended any changes to your lifestyle, any changes to your medications, or additional testing?

( ) Yes ( ) No

{IF ((above=Health care provider(s)) AND (above=Yes))}

What did your healthcare provider recommend? Please check all that apply. [ ] Lose weight
[ ] Stop smoking
[ ] Wear compression socks/stockings

[ ] Exercise more
[ ] Discontinue or change estrogencontaining contraceptive
[ ] Discontinue or change hormone replacement therapy
[ ] Exercise calf muscles or walk during travel
[ ] Take medication to prevent blood clots
[ ] Use heparin during pregnancy
[ ] Take baby aspirin
[ ] Use a blood thinner (anticoagulant) such as heparin or warfarin after surgery
[ ] Use a blood thinner (anticoagulant) such as heparin or warfarin for a longer duration [ ] Repeat F5 and F2 testing through your health provider
[ ] Have testing for other clotting disorders
[ ] Suggest relatives have testing for clotting disorders

[ ] Other:_____________
[ ] None of the above

{IF ((above=Health care provider(s)) AND (above=Yes) AND (above=Take medication to prevent blood clots))}

What medication did your healthcare provider recommend that you take to prevent blood clots? Please check all that apply.

[ ] Warfarin
[ ] Aspirin
[ ] Heparin
[ ] Another blood thinner [ ] I'm not sure

In the last 9 months, did you do any research on your own (online, at a library, or elsewhere) to learn about recommended changes to lifestyle, medications, or testing to reduce the risk of VTE?

[ ] Yes [ ] No

In the past 9 months, have you made any of the following changes as a result of your genetic risk for VTE? Please check all that apply.

[ ] Stopped smoking
[ ] Made effort to lose weight
[ ] Started wearing compression socks/stockings
[ ] Exercise more
[ ] Discontinued or changed estrogencontaining contraceptive
[ ] Discontinued or changed hormone replacement therapy
[ ] Started taking medication to prevent blood clots
[ ] Exercise calf muscles or walk during travel
[ ] Took baby aspirin
[ ] Took a blood thinner (anticoagulant) such as heparin or warfarin
[ ] Took a blood thinner (anticoagulant) such as heparin or warfarin after surgery
[ ] Took a blood thinner (anticoagulant) such as heparin or warfarin for a longer duration than

originally planned
[ ] Other:_____________ [ ] Made no changes

[If above=Took an oral or injectable blood thinner...]

What was the reason for starting an oral or injectable blood thinner? Please check all that apply. [ ] Diagnosed with a DVT or PE
[ ] Preventative measure after major surgery (requiring general anesthesia and lasting at least

30 minutes)
[ ] Preventative measure due to hospitalization or other prolonged immobility
[ ] Preventative measure due to physical trauma or injury
[ ] Preventative measure before or during longdistance travel (sitting in place for at least 8

hours, such as on a plane, bus or train)

[ ] Preventative measure due to pregnancy or recent childbirth
[ ] Diagnosis or treatment of cancer
[ ] Preventative measure due to use of a central venous catheter [ ] Other

[If female]
In the past 9 months, have you become pregnant?

( ) Yes ( ) No

(For those who answer yes to pregnancy question)
In the past 9 months, have you had any complications of pregnancy?
( ) Yes, I had a miscarriage (pregnancy loss at less than 20 weeks of gestation) ( ) Yes, I had a stillbirth (pregnancy loss at more than 20 weeks of gestation)
( ) Yes, I had intrauterine growth restriction or placental abruption
( ) No, my pregnancy has been uncomplicated

(For those who answer yes)
While pregnant in the past 9 months, have you taken any measures to prevent DVT or PE? Please check all that apply.
( ) Yes, I am drinking more water and making sure to move around frequently
( ) Yes, I am being closely monitored
( ) Yes, I am wearing compression socks/stockings
( ) Yes, I am taking low dose aspirin
( ) Yes, I am using heparin or low molecular weight heparin
( ) Yes, I am being managed by a high risk obstetrician/maternal fetal medicine specialist
( ) Yes, other: (free text)
( ) No, I am not doing anything differently

Which of your family members has had testing for genetic mutations associated with VTE? Please check all that apply.

[ ] Spouse or significant other [ ] Mother
[ ] Father
[ ] Sister(s)

[ ] Brother(s) [ ] Child(ren) [ ] Aunt(s)
[ ] Uncle(s)

[ ] Cousin(s)
[ ] Grandparent(s)

[ ] Other:_____________ [ ] None of the above

{IF any checked}

Did any of your relatives get genetic testing because of your results? ( ) Yes

( ) No
( ) I'm not sure

{IF any checked}

Did any of your relatives get genetic testing because another family member was diagnosed with VTE (a DVT / PE)?

( ) Yes
( ) No
( ) I'm not sure

{IF any checked}

Where were your relatives genetically tested?
( ) 23andMe
( ) Through a health care provider
( ) A combination of 23andMe and health care providers ( ) I'm not sure

PAGE: Awareness of Signs and Symptoms of VTE

Which of the following are associated with a higher than average risk of developing VTE? Please check all that apply.

[ ] Estrogen for birth control or hormonereplacement therapy [ ] Eating a high protein diet
[ ] Smoking
[ ] Family history of VTE

[ ] Obesity
[ ] Immobilization or sitting still for long periods of time [ ] Cell phone use
[ ] I'm not sure

If someone has one copy of the F5 or F2 mutation, which of their family members have an approximately 50% chance of also carrying it? Please check all that apply.

[ ] Mother [ ] Father

[ ] Sister
[ ] Brother
[ ] Halfsibling [ ] Child

What are the characteristic signs and symptoms of deep vein thrombosis (DVT, a blood clot in a deep vein)? Please check all that apply.

[ ] Pain in both legs
[ ] Pain in one leg
[ ] Forgetfulness
[ ] Swelling in one leg
[ ] Reddish or bluish skin discoloration [ ] Unusual warmth in one leg

[ ] Excessive sweating
[ ] Coughing up blood
[ ] Pain and swelling of both arms [ ] Pain and swelling in one arm
[ ] I'm not sure

What are the characteristic signs and symptoms of pulmonary embolism (PE, a blood clot in the lungs)? Please check all that apply.

[ ] Chest pain or discomfort [ ] Shortness of breath
[ ] Lightheadedness
[ ] Coughing up blood

[ ] Excessive sweating [ ] Sneezing
[ ] Rapid heartbeat
[ ] Wheezing

[ ] Sense of anxiety [ ] Forgetfulness
[ ] I'm not sure

PAGE: Attitudes Toward Genetic Knowledge

How important is it to you to know your genetic susceptibility for VTE? ( ) Very important
( ) Somewhat important
( ) Moderately important

( ) Slightly important
( ) Not at all important

Do you think that you can take steps to reduce the probability of VTE?

( ) Yes
( ) No
( ) I'm not sure

If you could do it all over again, would you choose to learn your genetic risk for VTE again? ( ) yes

( ) no
( ) I’m not sure
